# Food elimination, food substitution, and nutrient supplementation among ARV-exposed HIV-positive persons in southern Ghana

**DOI:** 10.1186/s41043-018-0157-x

**Published:** 2018-12-04

**Authors:** Amos K. Laar, Margaret Y. Lartey, Augustine Ankomah, Michael P. K. Okyerefo, Ernest A. Ampah, Demi P. Letsa, Priscillia A. Nortey, Awewura Kwara

**Affiliations:** 1 Department of Population, Family, & Reproductive Health, School of Public Health, University of Ghana, Accra, Ghana; 2 Department of Medicine, University of Ghana School of Medicine & Dentistry, University of Ghana, Accra, Ghana; 3 Department of Sociology, University of Ghana, Accra, Ghana; 4 Department of Epidemiology and Disease Control, School of Public Health, University of Ghana, Accra, Ghana; 5 Department of Medicine, Warren Alpert Medical School of Brown University, Providence, RI USA

**Keywords:** Nutrient supplementation, Food elimination, Food substitution, HIV, ART, Ghana

## Abstract

**Background:**

Optimal nutrition is a determinant of health in all persons. In persons living with HIV (PLHIV), nutrition is particularly important. Various factors, including dietary practices, play a role in guaranteeing nutritional health.

**Objectives:**

We investigated multiple non-prescription drugs use among HIV-positive persons receiving antiretroviral therapy (ART) from four treatment centers in southern Ghana. This paper, however, focuses on nutrient supplement use, food elimination, and food substitution practices by the PLHIV.

**Methods:**

Using quantitative and qualitative methods, we collected data from 540 HIV-positive persons at the health facility level. This paper focuses on only the quantitative data. Individual study participants were selected using a systematic random sampling procedure. Participants were interviewed after informed consent. We used univariate analysis to generate descriptive tabulations for key variables. Multivariable logistic regression modeling identified predictors of three practices (nutrient supplementation, food elimination, and food substitution). *P* value less than 0.05 or 95% confidence intervals facilitated determination of statistical significance. All analyses were performed using IBM SPSS Statistics for Windows, version 20.0.

**Results:**

The use of nutrient supplements was a popular practice; 72% of the PLHIV used various kinds. The primary motive for the practice was to boost appetite and to gain weight. A little over 20% of the participants reportedly eliminated certain foods and beverages, while 17% introduced new foods since their initial HIV diagnosis. All the three practices were largely driven by the quest for improved health status. We determined predictors of nutrient supplementation to be ART clinic location and having an ART adherence monitor. Having an ART adherence monitor was significantly associated with reduced odds of nutrient supplementation (AOR = 0.34; 95% CI 0.12–0.95). The only predictor for food elimination was education level (AOR = 0.29; 95% CI 0.30–0.92); predictors of food substitution were ART clinic location (AOR = 0.11; 95% CI 0.02–0.69) and anemia (defined as hemoglobin concentration less than 11.0 g/dl) (AOR = 0.21; 95% CI 0.12–0.85).

**Conclusions:**

The practice of supplementation is popular among this group of PLHIV. Food elimination and substitution are practiced, albeit in moderation. The predictors identified may prove helpful in provider-client encounters as well as local HIV programming.

## Introduction

Optimal nutrition is key to health. In the case of persons living with HIV (PLHIV), nutrition is particularly important. Nutrition can help boost immune function, maximize the effectiveness of antiretroviral therapy (ART), reduce the risk of other chronic diseases, and contribute to an overall quality of life [1]. Studies have shown that HIV-positive people with poor diets develop AIDS more quickly [2–5]. The many and multilayered factors that affect nutrition in HIV are at individual, household, and community levels. Individual-level factors include inherent metabolic changes and gastrointestinal disorders [6, 7], effects of various opportunistic infections [5], side effects of medications, and the effect of the HIV itself. Household-level factors are related to household attributes such as poverty and food insecurity [8]. Community-level factors relate to socio-political, religious, cultural, and structural factors [9, 10]. These factors can in a linear or in an interactive fashion impact negatively on the nutritional status of a PLHIV.

As other infections do, HIV increases metabolic rate. The body’ need for energy and protein is increased as it struggles to maintain optimal immune function and repair damaged cells [11–13]. The work of Mangili et al. also shows that HIV medications increase resting energy expenditure independent of viral load [14]. This translates into increases in nutrient requirements [15]. While increasing nutrient requirements, HIV infection at the same time can lead to reduced food intake. Several pathways explain this. First, reductions in food intake can result from painful sores in the mouth and/or esophagus. Side effects of the infection, such as fatigue, depression, and changes in mental state, can affect food intake as well. Further, specific nutrient deficiencies, which may be due to HIV infection, can affect a person’s appetite and interest in food. Side effects of medications, which include nausea, vomiting, diarrhea, abdominal cramps, can also result in suboptimal dietary intakes. A recent Ghanaian study that determined the daily intakes of some important nutrients by HIV-positive pregnant women revealed a high prevalence of inadequate dietary intake among those with nausea, vomiting, and oral lesions [16]. Another possible cause of reduced intake is food insecurity resulting from HIV-related factors [17, 18].

To therefore achieve optimal nutritional health, HIV-positive persons not only need to eat, they need to have access to diverse foods, consume adequate quantities, as well as be linked to optimal clinical care. In resource-constrained settings, however, many factors preclude these. As indicated earlier, the very potent weapon for HIV, antiretroviral (ARV) medications, is also one of the many clinical precipitators of reduced food intake. Although ARV efficacy in sustaining durable suppression of HIV replication, which in turn reduces rates of hospitalization, opportunistic infections, progression to AIDS, and death [19–21] are confirmed, side effects associated with their use can lead to compromised nutritional status [22]. Adverse side effects of ARVs are often related to the gastrointestinal system and include nausea, vomiting, diarrhea, abdominal cramps anemia, malabsorption, loss of appetite, and changes in taste of familiar foods. HIV-positive persons also end up with oral and esophageal lesions, sore throat, and swollen glands that result in a reduction of food intake. In an attempt to mitigate some of the adverse effects of ARVs, people often resort to herbs, potions, dietary supplements, and non-prescription remedies [23]. Some of the gastrointestinal side effects often drive people to make dietary modifications such as introducing new foods and/or eliminating others [1, 24, 25].

Like ARVs, some dietary supplements play important roles in the lives of many people living with chronic and often life-threatening medical conditions. However, there are concerns about their use. Such concerns generally stem from the potential for adverse interactions with conventional medicines and patients replacing evidence-based health care with untested remedies [26]. Studies show that the most common dietary supplements in PLHIV are aimed at “boosting immune functioning” such as mega-dose vitamins, and anti-oxidants, body cleansing products such as teas, and herbs such as ginseng [27]. Suffice it to say that many of these usually result in the elimination and/or restriction of certain foods. Others are contraindicated when used with antiretroviral medications [27, 28].

In addition to the clinical issues described above, there are social issues that affect the ability to acquire the kind of food needed to maintain optimal health among PLHIV. Food insecurity and HIV positivity have a cyclical relationship [29]. Being infected with HIV can limit productivity, leading, in turn, to loss of income while healthcare costs continue to increase [30]. The other factors include housing [31], challenges related to accessing ART [32], and food assistance [33].

While extensive research has been conducted among PLHIV to explore reasons why they may not adhere to ARV medication regimens [34–36], there is relatively little exploration, of whether they receive or adhere to nutritional recommendations from primary healthcare providers. There is equally little work done on the extent of food substitution or food elimination as a practice among PLHIV on ART. This paper assessed the extent and predictors of nutrient supplementation, the practice of food substitution or elimination among ARV-exposed PLHIV in southern Ghana.

## Methods

### Design and study sites

This study forms part of a larger original study that was conducted to examine non-prescription drug use among HIV+ persons on ART, adherence to ART, and barriers HIV+ persons face accessing ART in Ghana. A cross-sectional study using surveys and in-depth interviews was conducted to collect data from healthcare providers and a total of 540 adult HIV+ persons receiving ART at four treatment centers in the Eastern and Greater Accra regions of Ghana. For the purposes of the current paper, we focus on and report findings from the quantitative survey.

Surveys were conducted at four health facilities where ART is offered to HIV-positive clients. These health facilities are the Fevers Unit of the Korle Bu Teaching Hospital and the ART center at the Tema General Hospital (both in the Greater Accra region of Ghana). The Atua Government Hospital and St Martins de Porres Hospital ART centers were the other rural sites in the Eastern Region of Ghana. The Korle Bu Teaching Hospital is one of the tertiary hospitals in the southern part of Ghana. The Fevers Unit affiliated to the Department of Medicine, University of Ghana has the largest population of PLHIV at a single site. At the time of the study, the Fevers Unit of the Korle Bu Teaching Hospital had about 6000 patients on ART. Tema General Hospital (a regional hospital) had about 1500 HIV-positive clients who were enrolled on ART. The Atua Government Hospital and the St. Martins Hospital had about 4800 and 4000 HIV-positive clients on ART respectively.

### Sample size estimation, sampling, and summary of other field procedures

The sample size for the quantitative component of the study was determined using Statcalc in Epi Info 2000 package [37]. Both the population-based survey component and the unmatched cohort/cross-sectional study were used—first to provide at least 95% level of precision for estimating the prevalence of the three practices of interest (food elimination, food substitution, and nutrient supplementation) and second to provide power to detect predictors of these practices. Neither the exact proportions of the three key practices (nutrient supplementation, food elimination, and food substitution) nor the odds of engaging in the practices in relation to the potential predictors were known in this population. These were each assumed to be 50% but could be as low as 45% (worst acceptable level). The sample size from the guesstimated proportions was larger than that estimated using odds ratios. This was thus preferentially chosen. Thus, with an alpha of 0.05 and a statistical power of 90%, 434 clients was computed as the minimum sample size. This sample size was further increased by 20% (to account for contingencies such as non-responses or recording errors) and rounded up to 521 clients. However, being part of a larger study with an overall sample size of 540, all of the 540 PLHIV were used for this analysis.

Prior to sampling, the probability proportional to size weighting procedure was employed in the allotment of the PLHIV to the four study sites. Study participants were selected using a systematic random sampling with random start. To do this, the master list of ART clients at each facility served as the site-specific sampling frame. The sampling interval (*n*) for each site was derived by dividing the total number of participants on the monthly register by the required sample at each site. The total number of clients needed from a particular study site was interviewed between May 5 and June 30 2014.

Fieldwork was conducted by eight trained research assistants (RAs). RAs were recruited based on survey experience and knowledge of the local area. Training entailed introduction to the study objectives, goals, methods, and expected outcomes. There were extensive role-plays to ensure accuracy during fieldwork. Study tools were pretested after the training, and the needed validations done prior to the actual data collection.

With the help of health personnel managing the ART clinics, eligible study participants were identified and interviewed. The interview elicited information on the various medications and remedies used by the ART clients (ART and other approved allopathic and traditional/herbal medications, non-prescription drugs, nutrient supplements, etc.). Also recorded as part of the interview were potential predictors of the three study outcomes of interest such as “place of residence, sex, age, religious affiliation, level of education of respondent, and whether or not respondent had an adherence monitor.” Clients’ hospital records were also reviewed, and relevant data extracted. The interviews further assessed motivations for the various practices (problems with eating in general use of nutrient supplements, food elimination, food substitutions). Variables measured through lab-based equipment included body mass index (BMI) (kg/m^2^), CD4+ cell count, and hemoglobin concentration (g/dl). All such were determined using standard procedures.

### Data management and analysis

Interviews were done using paper-based questionnaires. Our data cleaning protocol required data collectors to review all completed data collection forms and correct errors/inconsistencies before hand-delivering them to field supervisors. Supervisors further reviewed the forms for accuracy, consistency, and completion. Once the data collection forms were considered complete, they were securely delivered to the principal investigator’s office where they were kept in locked filing cabinets. Measures instituted to address entry errors included hiring two competent and motivated data entry clerks, who then doubly entered data into pre-programmed data screens designed in CSPro (Census and Survey Processing System). Data were exported to IBM SPSS Statistics, version 20, for consistency checks and validation. Cleaned and validated data sets were analyzed using the same program.

We used univariate analysis to generate descriptive tabulations for key variables. To determine predictors of the key outcomes of interest (nutrient supplement use, food elimination, and food substitution), three independent multivariable logistic regression models were constructed using various background, socio-demographic, and clinical attributes of PLHIV as potential predictors.

Variables with *P* < 0.25, at the bivariate analysis (data not shown), or those previously reported to be associated with nutrient supplement use were selected into the multiple regression models. Several factors were considered potential predictors as per Table 2. A priori determined confounders introduced into the models included age, place of residence, and educational background. We employed a standard logistic regression modeling in SPSS (the “Enter” method) in our analysis. With this method, all the variables were entered into a full model generated in a single step. The attributes of the model are included in the tables presented. *P* value < 0.05 was used to denote statistical significance.

### Ethical considerations

Participation in the study conformed to the required ethical guidelines for use of human subjects. The study proposal was reviewed and approved by the Ethical Review Committee of the Ghana Health Service, Research and Development Division, Accra (Protocol ID NO: GHS-ERC 03/11/13). Permission was granted from the facilities within which the study was conducted. Informed consent was obtained from all participants after the objectives and the methodology of the study were explained to them. Participation in the study was completely voluntary, and no financial or material benefits were given. The privacy and confidentiality of every participant was ensured throughout the study period. Identification numbers (and not names) were used to disguise identity. Every member of the data collection and analysis team was cautioned during the training sessions to maintain strict confidentiality and anonymity of study data and participants. The participants, who were all adults (18 years or older), further consented to the publication of the study findings.

## Results

Table 1 presents the background, socio-demographic, and selected clinical characteristic of study participants. Close to half of the study sample (46%) was from the ART centers in the Greater Accra region; the remaining 54% were from the rural Eastern Region study sites. About three quarters (74%) of the respondents were female. Of 400 women who formed part of the study sample, data on physiologic state was available on 357. Of these, 3% were pregnant and 4% nursing young children. The sampled population was overwhelmingly Christian (90%). About 80% of them had some form of formal education. Close to 50% of the study participants either were anemic or had CD4 count of less than 350 cells/mm^3^. The prevalence of low body mass index/underweight (indicative of chronic energy undernutrition) was comparable to the prevalence of obesity among the study sample (Table 1).

**Table 1:** Background, socio-demographic, and selected clinical characteristic of study participants

ART/study site	Frequency	Percent
Atua Government Hospital	146	27.0
St Martin’s Martins de Porres Hospital	148	27.4
Tema General Hospital	93	17.2
Fevers Unit, Korle Bu Teaching Hospital	153	28.3
Place of residence		
Urban	275	50.9
Rural	265	49.1
Sex of respondent		
Male	140	25.9
Female	400	74.1
Religious affiliation of respondent		
Not religious	10	1.9
Christian	485	89.8
Muslim	43	7.9
Traditionalist	2	0.4
Respondent’s level of education		
No formal education	109	20.1
Primary	123	22.9
JHS	170	31.4
SHS/vocational	102	18.9
Post secondary/tertiary	36	6.7
Total	509	100.0
Age^1^		
18–19	5	0.9
20–24	12	2.2
25–35	126	23.3
36–60	374	69.3
61 or older	23	4.3
Does respondent have an adherence monitor		
Yes	293	54.5
No	245	45.5
Total	538	100.0
Physiologic status of female respondents		
Pregnant	10	2.8
Lactating	14	3.9
Not pregnant	333	93.3
Total	357	100.0
Body mass index (BMI)^2^ (kg/m^2^)		
Underweight (BMI < 18.50)	53	12.2
Normal weight (BMI 18.50–24.99)	236	54.1
Overweight (BMI 25.00–29.99)	99	22.7
Class I obesity (BMI 30.00–34.99)	29	6.7
Class II obesity (BMI 35.00–39.99)	11	2.5
Class III obesity (BMI > 40.00)	8	1.8
Total	436	100.0
CD4+ cell count^3^		
CD4+ cell count < 350	235	47.3
CD4+ cell count ≥350	262	52.7
Total	497	100.0
Hemoglobin concentration (g/dl)^4^		
Anemic < 11.0 g/dl	239	46.4
Normal/Hb ≥ 11.0 g/dl	276	53.6
Total	515	100.0

^1^Mean age is 42.3 age ranged from 18 to > 60 years

^2^Mean BMI is 23.9335 and ranged from 13.61 to 50.00; 104 of the cases have missing weight or height

^3^Median CD4 cell count is 373; range from 2 to 1663; 43 cases are missing CD4 measurements

^4^Mean Hb is 11.1 and ranged from 5.8–19.2; 25 cases are missing Hb measurements

**Fig 1:**
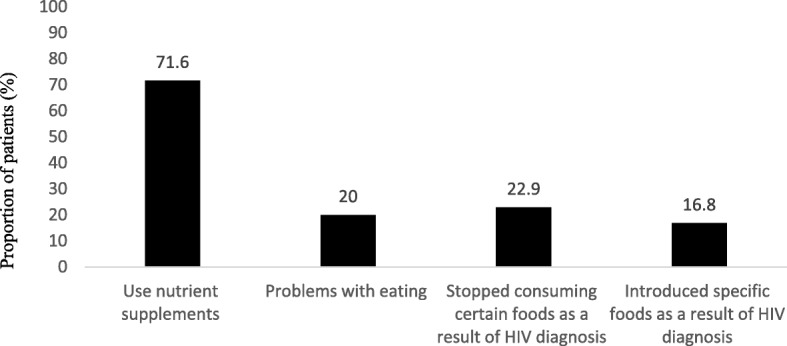
Nutrient supplement use, food elimination, and food substitution among PLHIV on ART

We present in Fig. 1 the extent of nutrient supplement use, food elimination, and food substitution among the study sample. Eighty percent of the PLHIV interviewed indicated having no problem with eating in general. The 20% with eating problems stated a number of reasons for their inability to eat properly. Lack of appetite/nausea/vomiting were the most common problems (mentioned 34 times), financial challenges were mentioned 12 times, and sore throat/pains in the mouth or stomach 11 times. Heart burn, a bitter taste in the mouth, tastelessness of food, and inability to afford the foods they want were some other reasons given. Some also complained of distention and pain in the abdomen when eating. To address this problem, some included more fruits and energy drinks in their diets. Many others began eating small meals at regular intervals and taking unidentified pills and tablets as well as “camphor tablets” to help with abdomen distention. Others forced themselves to eat despite their symptoms. Yet others resorted to nutrient supplementation.

Out of the total number of PLHIV interviewed, about 72% of them confirmed using nutrient supplements. These supplements include *Fersolate*, *folic acid*, *B-complex*, *Selevite*, and *multivitamin tablets*. The respondents mentioned other trade names of nutrient supplements such as *Vitafol*, *Bioferon*, *Astymin*, *Intravita*, *Selevite*, *Lutavita*, *Eleron*, *Zincovite*, *Zipferon*, *Zincolac*, and *Zincofer*. The primary reason survey respondents gave for taking supplements was to “enable them to eat well and to gain weight.” Majority of the respondents reported that they began to take supplements upon the advice of doctors, nurses, and other health workers.

A little over 20% of the interview participants reportedly eliminated certain foods and liquids since their HIV diagnosis. The foods or beverages eliminated included alcoholic beverages, fatty foods, palm oil, meat, milk, cocoyam fufu, and sugary foods. A variety of reasons were given by the respondents for these practices. Such included to control their blood pressure, reduce heart burn, reduce their protein level, live longer, and other undisclosed personal reasons. Others stopped upon the advice from fellow patients while others wanted relief from their symptoms such as stomach ache and upset, inability to sleep, diarrhea, vomiting, severe chest pain, uncomfortable feeling, and general feeling of weakness. As with nutrient supplement use, many indicated that they stopped certain foods upon consulting with nurses, doctors, and health workers who advised them to stop since it suppresses the potency of the ARV and reduces the effectiveness of the ART.

Out of a total of 471 PLHIV who responded to the question on food substitution, only 16.8% had introduced new foods after their initial HIV diagnosis. These foods were mainly fruits (mango, banana, watermelon, orange, pawpaw, carrots, pear, apple), vegetables (spinach leaves or kontomire), “tom brown” (a local cereal bran made from maize, sorghum, and groundnuts), “fufu,” palm nut soup, and dry fish. The primary reason respondents gave for introducing these new foods in to their diet was health worker (doctors or nurses) recommendation. Other reasons given were that it improved their nutritional status, boosts the immune system to fight off opportunistic infections, and “gives them blood” and enough strength to work.

We constructed three independent multivariable regression models to predict the practices of nutrient supplementation (model 1), food elimination (model 2), and food substitution (model 3). The models identified predictors of the stated outcomes after adjusting for a number of covariates.

In model 1, ART clinic location and having an ART adherence monitor were predictive of nutrient supplement use. Thus, compared to ART service users from the Korle Bu Fevers Unit (a teaching hospital setting), those from the Eastern Region ART clinics were significantly less likely to use nutrient supplements. On the contrary, PLHIV from the Tema General Hospital (a district hospital located in the city) had nine times to use nutrient supplements (adjusted odds ratio [AOR] = 8.99; 95% CI 2.37–34.17) (Table 2). Thus, having an ART adherence monitor was significantly associated with reduced odds of nutrient supplement use (AOR = 0.34; 95% CI 0.12–0.95).

**Table 2:** Background, socio-demographic, and clinical correlates of nutrient supplement use, food elimination and substitution

Attribute	Nutrient supplement use *Model 1*			Eliminated certain foods due to HIV diagnosis *Model 2*			Introduced new/substituted foods due to HIV diagnosis *Model 3*		
	AOR	95% CI		AOR	95% CI		AOR	95% CI	
		LB	UB		LB	UB		LB	UB
ART site									
Atua ART clinic	0.26	0.06	0.61	0.38	0.11	0.95	0.20	0.03	1.20
St Martins de Porres Hospital	0.20	0.05	0.87	0.37	0.10	0.74	0.11	0.02	0.69
Tema General Hospital	8.99	2.37	34.17	0.99	0.17	5.78	0.16	0.02	1.35
Korle Bu Fevers Unit	1.00			1.00			1.00		
Sex of respondent									
Male	0.39	0.03	4.57	1.83	0.12	28.44	10.23	0.95	71.69
Female	1.00			1.00			1.00		
Place of residence									
Urban	2.37	0.71	7.91	0.55	0.22	1.41	0.76	0.24	2.48
Rural	1.00			1.00			1.00		
Respondent’s level of education									
No formal education	1.49	0.16	14.20	0.29	0.03	0.92	0.40	0.04	4.17
Primary	1.02	0.11	9.22	0.20	0.02	1.70	0.67	0.07	6.35
JHS	1.52	0.17	13.73	0.21	0.02	1.85	0.50	0.05	4.86
SHS/vocational	0.82	0.07	9.22	0.16	0.02	1.58	0.68	0.06	7.65
Post secondary/tertiary	1.00			1.00			1.00		
Does respondent have an adherence monitor									
Yes	0.34	0.12	0.95	1.09	0.36	3.25	0.65	0.16	2.66
No	1.00			1.00			1.00		
Physiologic state of female respondent									
Pregnant/lactating	1.51	0.67	3.41	1.49	0.77	2.85	1.38	0.68	2.80
Not pregnant	1.00			1.00			1.00		
Age categories									
< 35	0.85	0.41	1.78	1.17	0.58	2.33	1.22	0.52	2.85
≥ 35	1.00			1.00			1.00		
BMI									
Underweight	1.24	0.42	3.70	1.33	0.49	3.56	1.85	0.58	5.87
Not underweight	1.00			1.00			1.00		
CD4+ cell count									
CD4+ cell count < 350	1.08	0.54	2.17	0.83	0.44	1.54	1.12	0.54	2.33
CD4+ cell count ≥ 350	1.00			1.00			1.00		
Anemia status									
Normal/Hb ≥ 11.0 g/dl	0.75	0.36	1.56	0.89	0.47	1.67	0.47	0.21	0.85
Anemic < 11.0 g/dl	1.00			1.00			1.00		

*Abbreviations*: *AOR*, adjust odds ratios with accompanying 95% confidence intervals were determined using multiple logistic regression (all variables in this table were included in the model)

Nutrient supplement use model: Model summary Cox and Snell *R*^2^ (0.176), Nagelkerke *R*^2^(0.264), −2 Log likelihood 220.832 (estimation terminated at iteration number 20 because maximum iterations have been reached)

Food elimination model: Model summary Cox and Snell *R*^2^ (0.125), Nagelkerke *R*^2^ (0.180), −2 Log likelihood 264.135 (estimation terminated at iteration number 20 because maximum iterations have been reached)

Model for food substitution: Model summary Cox and Snell *R*^2^ (0.109), Nagelkerke *R*^2^ (0. 176), −2 Log likelihood 198.231 (estimation terminated at iteration number 6 because parameter estimates changed by less than 0.001)

For model 2, only one of the ten potential predictors—respondent’s level of education—significantly predicted food elimination. Those with no formal education were less likely to eliminate foods from their usual menus compared to those with post secondary/tertiary level of education (AOR = 0.29; 95% CI 0.03–0.92).

Model 3 aimed to identify predictors of food substitution/introduction of new foods, and confirmed ART clinic location and anemia (defined as hemoglobin concentration less than 11.0 g/dl) to be independent predictors of the practice. Anemic PLHIV were less likely to introduce new foods (AOR = 0.45; 95% CI 0.21–0.85). Also compared to the Korle Bu study site, PLHIV from the Atua Government Hospital (district hospital setting in a rural area), the St Martin’s (a rural sub-district hospital setting), and the Tema General Hospital study sites had lower odds of introducing new foods, although only the St Martins ART site retained its predictive power after controlling for nine other potential predictors (AOR = 0.11; 95% CI 0.02–0.69) (Table 2).

## Discussion

This paper presents and discusses the extent of nutrient supplement use, food elimination, and food substitution practices among PLHIV on ART in Southern Ghana. Our data show that majority (72%) of the PLHIV were users of nutrient supplements; 23% had eliminated certain foods and beverages upon HIV diagnosis, and 17% had substituted/introduced new foods post their HIV diagnosis. Significant correlates of nutrient supplement use included location of ART clinic and ART adherence monitoring. Predictors of food elimination were location of ART clinic and level of education. Anemia (defined as hemoglobin concentration < 11.0 g/dl) was the only significant predictor of the practice of food substitution. These key findings are discussed.

Our data show that 55% of the study respondents had an ART adherence monitor at the time of the survey and having an ART adherence monitor was significantly associated with reduced odds of nutrient supplement use (AOR = 0.34; 95% CI 0.12–0.95). Before discussing our key study findings, it would be useful to present background to adherence monitoring. At the time of the study, local guidelines relating to “ARV treatment initiation”—in line with WHO guidelines—contained both medical/clinical and non-medical eligibility criteria. Medical eligibility criteria included patients with CD4 count less than 350 cells/ml and/or the patient is symptomatic with HIV infection in WHO clinical stages III and IV and patient presents with severe hepatic liver function tests > 5 times the upper limit of normal, or end-stage renal disease; a patient having an acute opportunistic infection is not eligible to initiate ART. The acute opportunistic infections must be treated before initiation of antiretroviral therapy to avoid, for example, immune reconstitution syndrome. Therefore, service providers would provide appropriate adherence counseling and initiate ART for those who are eligible. The non-medical eligibility criteria included “do not initiate treatment, if treatment is not sustainable,” for example if the person is not able to cope with follow-up visits or facility is unable to assure continuity of care and if HIV+ is found to be unlikely to comply/adhere, PLHIV. For these HIV+ as well as not who are screened off with the medical eligibility criteria, service providers provide appropriate counseling and defer initiation into ART. For those who meet enrollment criteria and are enrolled into ART, adherence monitoring is a life-long initiative, just as ART. Due to limited trained personnel and socio-cultural limitations, this service may be delivered by trained health services if their relationship with HIV+ and proximity will facilitate regular interaction; these are also delivered by family members, friends, religious leaders, or other confidants of the HIV+. In our study, we aimed to assess the association of “service continuity adherence monitors/counsellors” and the three study outcomes (nutrient supplementation, food elimination, food substitution), and not receipt of adherence counseling during initiation into ART (as all HIV+ do receive). The variable was self-reported—in response to a survey question on whether or not HIV+ currently had an adherence counselor/monitor. That 45% of the PLHIV on ART did not have adherence monitors that could be interpreted as adherence monitoring continuity relapse. This is a phenomenon worth investigating in a separate follow-up study.

### Nutrient supplementation

We found a high prevalence of micronutrient supplementation by the survey participants, and this is in keeping with previous research studies [38–42]. These studies not only report on the extent of nutrient supplement use by PLHIV, they also report on several benefits of supplementation in PLHIV. Kaiser et al. in 2006 established that micronutrient supplementation in PLHIV on ARTs had an increase in CD4 cell count and the supplements were generally well tolerated. Nutrient supplementation in African women with HIV significantly improved CD4 count and had a sustained effect for a median of 5 years in the population studied [43]. Bormann et al. in their study reported commonly used nutrient supplements to be mega-dose vitamins, anti-oxidants, teas, and herbs. These were primarily aimed at boosting immune function [27]. Majority of the respondents in the current study reported such trade names as Selevite, Zincovite, and Zincolac. Two main reasons were given in support of nutrient supplementation—“to increase appetite” and “to gain weight.” Generally, nutrient supplements were well tolerated and no adverse clinical effects were reported among participants who took supplements. The high usage of nutrient supplement in our study agrees with many [38–42], but not all studies. Bukusuba et al. observed in their study conducted among HIV women in Eastern Uganda that only about 20.3% of them added nutrient supplement to their regular diets [44]. Work done by Vorster et al. and Holcomb did link urbanization to an increase in micronutrient uptake and consumption of diverse food categories [45, 46]. Kalichman et al. reported that 69% of HIV-positive men used complementary medicine products and practices [47]. Fawzi et al. in their research among Tanzania PLHIV hinted that supplementation may be an important yet, inexpensive prophylactic and therapeutic measure for HIV-1-infected people [48]. Deficiencies of vitamins A, E, B_6_ B_12_, zinc, and selenium have been shown to have adverse clinical outcomes during HIV infections and a higher mortality rate [43, 49]. HIV affects nutritional status from the onset of infection and in all stages of the disease [38, 50, 51]. HIV progressively weakens the immune system, leading to opportunistic infections. Infections cause and exacerbate poor nutrition. Poor nutrition makes it impossible for PLHIV to stay healthy and productive much worse if this state of poor nutrition pre-exists. To offset such undesirable clinical as well as physical manifestations, PLHIV turn to alternative and complimentary medications: among which are herbs and nutrient supplement [52, 53].

According to available research, WHO recommends a daily dose of the recommended nutrient intake (RNI) for all micronutrients [54], especially for PLHIV. The current study looked primarily at the prevalence of nutrient supplementation, although and earlier Ghanaian study in the setting of the current study reported suboptimal intake of various nutrients among female PLHIV [16]. Further exploration is needed to determine if our research population meets this recommended standard in the dosing, amount, and quality of their micronutrient supplementation. Our data also show that about one out of three of the respondents took other non-prescription remedies including herbal concoctions (data not shown). Although these were named, our research did not examine their chemical composition. Several studies [26, 27, 47, 55, 56] have reported on micronutrient supplementation [55, 56], and its adverse interaction with prescription drugs like ARVs have also been put forth [26, 27, 47]. It remains entirely possible that PLHIV on ARVs in our geographic area might suffer adverse consequences due to their concurrent use of prescription medications and non-prescription herbal supplements of unknown chemical composition. The study determined significant correlates of non-prescribed nutrient supplement use to include location of ART clinic. Our collective experience supports this finding. We believe that practices by service providers and service users in a cosmopolitan city/teaching hospital versus those at the district level will differ in many respects. The ART clinic at the Korle Bu Teaching Hospital is manned by a team highly specialized physicians, pharmacists, and clinical psychologists. Second, the clientele of the clinic are likely to be better placed to reprimand and negative feedback for services given outside orthodox practices and treatment guidelines. This is in contrast to clinics run by nurses who with patients are one large family and are more tolerant to certain behaviors. Of note, the model could explain about 26% of the variability in the practice (Nagelkerke *R*^2^ = 0.26; Table 2).

### Food elimination and food substitutions

Majority (80%) of respondents in this study had no problem with eating. Nevertheless, being HIV positive and on ART did prove to be problematic for some of the respondents. The HIV state paradoxically causes heightened nutritional need but early satiety in individuals [24]. This compounded with clinical manifestations of oral lesions, sore throat, esophageal infections, and malabsorption impede and impair swallowing of food [24]. A study by Laar et al. among HIV-positive pregnant adolescents in Ghana corroborates the observation in this study. Their study found out that participants with oral lesions or experiencing nauseate feeling and vomiting had a very low intake of nutrients compared to the others who had no such complications. Such feeling can precipitate food elimination or substitution.

In addition to the above, people on ARVs tend to suffer from adverse side effects of the medications that are mainly related to the gastrointestinal system such as diarrhea, vomiting, upset stomach, and nausea. These adverse medication effects can itself lead to compromised nutritional status [22]. Many PLHIV therefore are unable to ingest food while experiencing these adverse effects of ARVs.

Almost a quarter of the respondents reported that they experienced gastrointestinal adverse effects related to their ARV medications and/or certain foods that they consumed. Examples of foods that had been eliminated were fatty foods, starchy foods, and some animal protein sources. Care however needs to be taken since these self-reported food eliminations could affect their overall nutritional status over the long term. To the best of our knowledge, these food categories that the respondents had self-eliminated had not been replaced by options that were better tolerated; therefore, there is the risk of an unbalanced diet that does not have adequate proportions of all food groups. As far as we know, no studies have explored the long-term nutritional effects of food elimination by PLHIV based solely on adverse side effects and not on evidence-based health professional advice.

PLHIV on ARVs have an increased risk for developing lipodystrophy and associated metabolic problems, including dyslipidemia and insulin resistance [57]. This may increase their risk for chronic health problems such as coronary heart disease and diabetes. Lifestyle modifications, healthy diet, and regular exercise have been shown to significantly reduce the risk of these chronic diseases. In our study, certain food eliminations were based on healthy lifestyle and wellbeing choices. Respondents reported that they had given up certain food items in order to “feel better, sleep well, decrease their blood pressure, and live longer.” Many others reported that they presently refrain from alcohol, tobacco, and cigarettes. These overall lifestyle changes could increase the overall prognosis of PLHIV.

One of the objectives of this paper was to explore whether PLHIV receive and/or adhere to nutritional recommendations from primary care providers. Nearly 20% of them had introduced specific foods post HIV diagnosis. Many participants reported that they had eliminated certain foods from their diet based on health professional advice that it was contraindicated with ARV use and/or suppressed ARV effectiveness. They also confirmed that many foods had been added on because of health professional advice. In a study by Nti et al. [58] among PLHIV in Ghana, it was found that majority had fair to adequate knowledge of healthy nutritional options; however, this did not translate into a higher quality of diet and overall nutritional status. This gap between knowledge and practice can be linked to social and cultural determinants related to food insecurity. Many of the foods added were fruits, vegetables, proteins, and plant-based highly nutritional foods such as sorghum, groundnuts, and millet. It is therefore a matter of concern that majority of the respondents, although having adequate knowledge of nutrition, are unable to add these healthier food options to their current diet. Of note, the practices reported are not entirely attributed to the HIV disease or the side effects of the ARVs. Socio-cultural, religious, and economic factors may motivators. A recent Ghanaian study [59] reported that the family, culture, and the economic status of a household influence food choices and portion sizes. Sztam et al. have explained that the economic strain causes poor quality of life and decreased work productivity of families and individuals, thus forcing them to opt for cheaper food choices [60]. In the current study, location of ART clinic and level of education significantly predicted food elimination practices, but not the studied religious or socio-cultural variables.

## Conclusions and recommendations

The use of nutrient supplements is popular among this group of PLHIV. Food elimination and substitution are practiced, albeit in moderation. The only significant predictor for food elimination was education level, whereas ART clinic location and anemia (defined as hemoglobin concentration less than 11.0 g/dl) significantly predicted food substitution behaviors. The identified predictors may prove helpful in provider-client encounters as well as local HIV programming. Given the association between clinic location and ability to substitute health-promoting foods, patients and care providers at rural sites may need education about potential health-promoting benefits of the practice. Efforts at providing and scaling-up ART to all HIV+ persons ought to recognize these food and nutrition-related dynamics and thus embed interventions that address them. Among others, we recommend the institutionalization of individualized nutrition assessment and care and long-term investment to improve nutrition literacy of both care providers and their clients.

## Abbreviations

AIDS: Acquired immune deficiency syndrome
ART: Antiretroviral therapy
ARV: Antiretroviral
CSPro: Census and Survey Processing System
HIV: Human immunodeficiency virus
PLHIV: Persons living with human immunodeficiency virus
RAs: Research assistants
SPSS: Statistical Package for the Social Science
TB: Tuberculosis
WHO: World Health Organization
